# Green Tea Extract Enrichment: Mechanical and Physicochemical Properties Improvement of Rice Starch-Pectin Composite Film

**DOI:** 10.3390/polym14132696

**Published:** 2022-06-30

**Authors:** Wantida Homthawornchoo, Jaejoon Han, Pimonpan Kaewprachu, Orapan Romruen, Saroat Rawdkuen

**Affiliations:** 1 Food Science and Technology Program, School of Agro-Industry, Mae Fah Luang University, Chiang Rai 57100, Thailand; orapan.rom13@lamduan.mfu.ac.th; 2 Innovative Food Packaging and Biomaterials Unit, Mae Fah Luang University, Chiang Rai 57100, Thailand; 3 Department of Food Bioscience and Technology, College of Life Sciences and Biotechnology, Korea University, Seoul 02841, Korea; jjhan@korea.ac.kr; 4 College of Maritime Studies and Management, Chiang Mai University, Samut Sakhon 74000, Thailand; pimonpan.k@cmu.ac.th; 5 Cluster of Innovative Food and Agro-Industry, Chiang Mai University, Chiang Mai 50100, Thailand

**Keywords:** green tea extract, rice starch, pectin, active film, antioxidant

## Abstract

The effects of green tea extract (GTE) at varying concentrations (0.000, 0.125, 0.250, 0.500, and 1.000%, *w/v*) on the properties of rice-starch-pectin (RS-P) blend films were investigated. The results showed that GTE addition enhanced (*p* < 0.05) the antioxidation properties (i.e., total phenolic content, DPPH radical scavenging activity, and ferric reducing antioxidant power) and thickness of the RS-P composite film. The darker appearance of the RS-T-GTE blend films was obtained in correspondence to the lower L* values. However, the a* and b* values were higher toward red and yellow as GTE increased. Though GTE did not significantly alter the film solubility, the moisture content and the water vapor permeability (WVP) of the resulting films were reduced. In addition, the GTE enrichment diminished the light transmission in the UV-Visible region (200–800 nm) and the transparency of the developed films. The inclusion of GTE also significantly (*p* < 0.05) lowered the tensile strength (TS) and elongation at break (EAB) of the developed film. The FT-IR spectra revealed the interactions between RS-P films and GTE with no changes in functional groups. The antimicrobial activity against *Staphylococcus aureus* (TISTR 764) was observed in the RS-P biocomposite film with 1% (*w/v*) GTE. These results suggested that the RS-P-GTE composite film has considerable potential for application as active food packaging.

## 1. Introduction

Demand for innovative and improved biodegradable packaging has rapidly increased over the years [1] due to consumer awareness and concerns about food safety [2,3], limited resources [4], and environmental impacts [5,6]. The biocompatible [7], biodegradable [8], nontoxic, and sustainable [9] packaging materials are especially attractive for further development to address these concerns [2,10]. As a result, the development of active packaging materials has moved toward the adoption of natural active ingredients [11,12,13].

Rice starch (RS) is known for its abundance, low cost [14], and renewability [15]. It is one of the potential biopolymers for manufacturing edible and biodegradable films. Amylose and amylopectin are the polysaccharides found in rice starch that mainly contribute to the gas barrier, and optical and mechanical properties in the RS-based films [15]. However, owing to its inherent hydrophilicity, rice-starch-based film has limited industrial application [16] due to its impractical mechanical properties, especially at high humidity, and the lack of an efficient barrier against high-polarity compounds [17,18,19]. This constraint has motivated researchers to pursue different approaches to improve the rice-starch-based film properties, including modification of starch properties [20] and incorporation with other biomaterials [21], to overcome the problem to increase its chance of usability for a specific application [17,22].

Pectin (P) is an entirely biodegradable, biocompatible, and edible biopolymer for food packaging material [23,24]. The addition of pectin as a co-biopolymer is particularly interesting as pectin can act as a carrier of the bioactive ingredient [25]. Pectin also has a good film-forming property [26] and helps improve the mechanical and optical properties of the pectin composite film [10,27,28]. Moreover, pectin can be obtained as a by-product of the agricultural and food industry [29,30].

The incorporation of natural antioxidants into packaging materials has become popular as oxidation is one of the major problems affecting food quality [31]. This approach can also reduce the use of synthetic food additives in food products [32]. Natural antioxidants and antimicrobial agents are alternatives to synthetic food preservatives to avoid potential health concerns and a strict regulation associated with the usage of the artificial food preservatives [31,33]. The inclusion of green tea extract (GTE) has been extensively studied to improve the functional properties of various film bases, for example, polypropylene [34], agar-gelatin [35], gelatin [36], surimi [37], and starch-LLDPE [38], poly(vinyl alcohol) (PVA) [39]. Green tea (*Camellia sinensis* L.), a non-fermented tea, is rich in polyphenolic compounds, especially catechins. The catechin exhibits potent antioxidant and antimicrobial activities [31,32,35,39]. Not only can GTE-enriched biopolymer edible film extend the shelf-life and improve the quality of food products, but it may also have potential health benefits for the consumer [35].

Though green tea extract has been studied on various polymer films, there has been no report on all plant-based biopolymers such as rice starch and pectin composite film (RS-P). This research focused on utilizing low-cost, petroleum-free, biodegradable, plant-based rice starch and pectin composite film. Therefore, the effect of green tea extract on the physicochemical, mechanical, optical, and barrier properties of the RS-P composite films was investigated. In addition, the antioxidant and antimicrobial properties of the RS-P composite film infused with GTE were also examined.

## 2. Materials and Methods

### 2.1. Materials

Native rice starch was purchased from Thai Flour Industry Co., Ltd., Bangkok, Thailand. Dried green tea leaves were purchased from Thai Tea Suwirun Co., Ltd., Chiang Rai, Thailand. Folin–Ciocalteu’s phenol reagent and gallic acid (≥99%) were purchased from Fluka (Buchs, Switzerland). DPPH (2,2-diphenyl-1-picryhydrazyl) and TPTZ (2,4,6-tripyridyl-s-triazine) were purchased from Sigma-Aldrich (St. Louis, MO, USA). All other chemicals and reagents used in this study were of analytical grade.

### 2.2. Preparation of Green Tea Extract

Green tea extract (GTE) was prepared according to the previous report with slight modification [40]. In brief, dried green tea leaves were brewed with hot water of 95 °C at a 1:20 ratio for 20 min with constant stirring. The brewing was carried out twice under the same conditions (once with 150 mL of hot water and another time with 100 mL of hot water). The mixture was filtrated through a plain muslin cloth to remove the green tea residues and evaporation at 40 °C using a rotatory evaporator (115 VAC, Cole-Parmer, Vernon Hills, IL, USA). The filtrate was freeze-dried and stored at –20 °C until further use.

### 2.3. Preparation of the RS-P Composite Film

The film-forming solution (FFS) was prepared using the air-dried-casting method mentioned by Kaewprahu et al. [41] with some modifications. Briefly, a mixture of rice starch (RS) and high-methoxyl pectin (P) at a 1:1 ratio was added to the DI water at 4% (solid *w/v*) with 30% (glycerol *w/w* of solid content). The FFS solution was subjected to heat at 85 °C for 30 min. The GTE at 0.000, 0.125, 0.250, 0.500, and 1.000 %*w/v* was added to the FFS after cooling to 40 °C. The FFS solution (4 ± 0.01 g) was then cast on a 50 × 50 mm^2^ silicone mold and dried at room temperature for 24 h. The RS-P composite films with GTE were peeled off and conditioned at 50 ± 5% relative humidity (RH), 25 ± 0.5 °C for 48 h, using the humidity dry cabinet (AH-80, Patron, Taichung City, Taiwan) before film characterization.

### 2.4. Film Property Determination

#### 2.4.1. Film Thickness

The thickness of the GTE-infused RS-P composite films was measured at nine random locations using a micrometer (Mitutoyo Corporation, Tokyo, Japan). Five film specimens were used to calculate the film thickness.

#### 2.4.2. Film Appearance and Color

The appearance of all films was taken by a Fujifilm FinePix S4900 digital camera (Fujifilm Thailand Co. Ltd., Bangkok, Thailand).

The color of the films was measured in the CIE system using a Color Quest XE (Hunter Lab, Reston, VA, USA). The standard white tile (L* = 97.55, a* = –0.03, and b* = 1.73) was used to calibrate the colorimeter prior to measurement. The color values of the developed films in terms of the lightness (L*), redness (a*), and yellowness (b*) were the average of the five film specimens.

#### 2.4.3. Film Transmittance and Transparency

The transmittance (%T) of the films in the Ultraviolet-Visible (UV-Vis) region (200–800 nm) was measured [41]. Briefly, the square film of 40 mm × 40 mm in size was placed into the cell holder of a UV-Vis spectrophotometer (Genesys 10S UV-Vis, Madison, WI, USA). The transmittance value recorded in triplicate at 600 nm was used to determine the light transparency. The film transparency was determined according to the following Equation (1):

Transparency = –log T_600_/*x* (1)

where T_600_ is the transmittance value of the film at 600 nm (%) and *x* is film thickness (mm).

#### 2.4.4. Film Moisture Content and Solubility

The moisture content (MC) of the film was determined using 2 cm × 2 cm film following the AOAC standard methods [42]. Moisture content was determined in triplicate and calculated using the percent weight loss from the initial weight. Briefly, a film sample of 20 mm × 20 mm in size was weighed and subjected to drying at 105 °C for 24 h. The moisture content of each film treatment is expressed as the percentage weight difference of the film before and after drying, as shown in the following equation (Equation (2)):

Moisture content (%) = ((Wi–Wf))/Wi) × 100(2)

where Wi is the initial weight of the film before drying and Wf is the final weight of the film after drying.

The film solubility (FS) was carried out in triplicate according to a method detailed by Kaewprachu et al. [41] and expressed as the percent weight loss from initial dry matter weight and the weight of dry matter of undissolved debris.

#### 2.4.5. Mechanical Properties

Tensile strength (TS) and elongation at break (EAB) were determined using a Universal Testing Machine (Lloyd Instruments Ltd., Fareham, Hampshire, UK). Films were cut into the 2 cm × 5 cm specimens. The initial grip length was set at 30 mm, and the cross-head speed used was 30 mm/min. Ten film specimens were tested for each film treatment.

#### 2.4.6. Water Vapor Permeability

Water vapor permeability (WVP) measurement was conducted in accordance with a modified ASTM method [43]. WVP cups were filled with silica gel (0% RH), covered with the film sample, and sealed using an O-ring and silicone vacuum grease. The cups were placed in a dry cabinet (AH-80, Patron, Taichung city, Taiwan) at 25 °C and 50% RH. The weight of the cups was carefully taken every hour for 8 h. The WVP of the film was expressed as g m/m^2^ s Pa. The WVP test was carried out in triplicate for each film treatment.

#### 2.4.7. Fourier Transform Infrared Spectroscopy Analysis

The FTIR Spectrum GX (PerkinElmer Inc., Waltham, MA, USA) was used to analyze the FTIR spectra of the films. Each film was examined in triplicate at 25 °C using the spectrum range of 4000 to 650 cm^–^^1^ with 64 scans and a resolution of 4 cm^–^^1^ [41].

#### 2.4.8. Determination of Total Phenolic Content

Prior to determination, the film extract solution of each film treatment was prepared. Briefly, 25 mg of the film was dissolved in 2.5 mL of distilled water and shaken for 3 h at 25 °C at 250 rpm. After that, the mixture was centrifuged at 3000× *g* for 10 min at 25 °C to collect the supernatant and used to determine total phenolic content (TPC), DPPH radical scavenging activity (DPPH), and ferric reducing antioxidant power (FRAP).

The TPC was determined in triplicate using the Folin–Ciocalteu assay [44]. The standard curve of the gallic acid in the specific concentration range of 20–100 µg/mL was constructed. The gallic acid equivalent (GAE) represented the total phenolic compound. The TPC is expressed as mg GAE per g dry weight of the film sample.

#### 2.4.9. Determination of DPPH Radical Scavenging Activity

The DPPH radical scavenging activity of the RS-P film containing GTE was investigated [41,45]. Briefly, 1.5 mL of the film extract solution was combined with 1.5 mL of 0.15 mM of DPPH in 95%-ethanolic solution, stirred vigorously, and let to stand in the dark for 30 min. The 95% methanol was used as the blank solution. The absorbance at 517 nm of the resulting solution was determined using the UV-Vis spectrophotometer (Genesys 10S UV-Vis, Madison, WI, USA). A standard curve of 10–60 μM Trolox was created. The DPPH antioxidant activity was evaluated and reported as µmol Trolox equivalents/g dried film.

#### 2.4.10. Determination of Ferric Reducing Antioxidant Power

The Ferric Reducing Antioxidant Power (FRAP) of each film treatment was acquired [45,46]. Briefly, the working FRAP reagent was prepared by blending 300 mM acetate buffer (pH 3.6), 10 mM TPTZ (2,4,6-tripyridyl-s-triazine) in 40 mM HCl solution, and 20 mM FeCl_3_·6H_2_O solution at a ratio of 10:1:1. To determine the FRAP value, the film extract solution (150 μL) was combined with the working FRAP solution (2850 μL) and then incubated in the dark for 30 min in a water bath (37 °C). The absorbance of the obtained color product (Ferrous tripyridyltriazine complex) was measured at 593 nm using the UV-Vis spectrophotometer (Genesys 10S UV-Vis, Madison, WI, USA). The standard curve of ferrous sulfate (0–1000 μM) was prepared. The FRAP values were expressed as µmol ferrous sulfate (Fe(II)) equivalents/g dried film.

#### 2.4.11. Antimicrobial Activities Test

Antimicrobial activities of the RS-P composite films enriched with GTE were carried out in triplicate using the agar disc diffusion method [41]. The developed films were tested against a Gram-negative bacteria (*Escherichia coli* TISTR 527) and a Gram-positive bacteria (*Staphylococcus aureus* TISTR 746). Briefly, all bacterial strains were cultured in a Mueller-Hinton (MH) (Difco, Detroit, MI, USA) broth and incubated in the shaker at 37 °C for 24 h. The bacteria were then streaked onto the MH agar plates and further incubated at 37 °C for 24 h to obtain a single colony. The optical density of the cultures was adjusted to 0.5 McFarland turbidity standards with 0.85% normal saline and then inoculated on MH agar plates using a sterile swab. Each film sample of 6 mm in diameter was sterilized with UV light for 30 min, placed on the inoculated MH agar plates, and incubated at 37 °C for 18–24 h. Ampicillin (10 μg/disc) was used as a positive control. The antimicrobial activities were observed through the inhibition zones.

### 2.5. Statistical Analysis

Data were expressed as mean ± standard deviation. The one-way analysis of variance (ANOVA) was performed by using an SPSS package (SPSS 23.0 for mac, SPSS Inc., Chicago, IL, USA). Duncan’s Multiple Range Test at a confidence level of 95% (*p* < 0.05) was used to determine the significant difference.

## 3. Results

### 3.1. Film Appearance, Color, Transmittance, and Transparency

**Figure 1:**
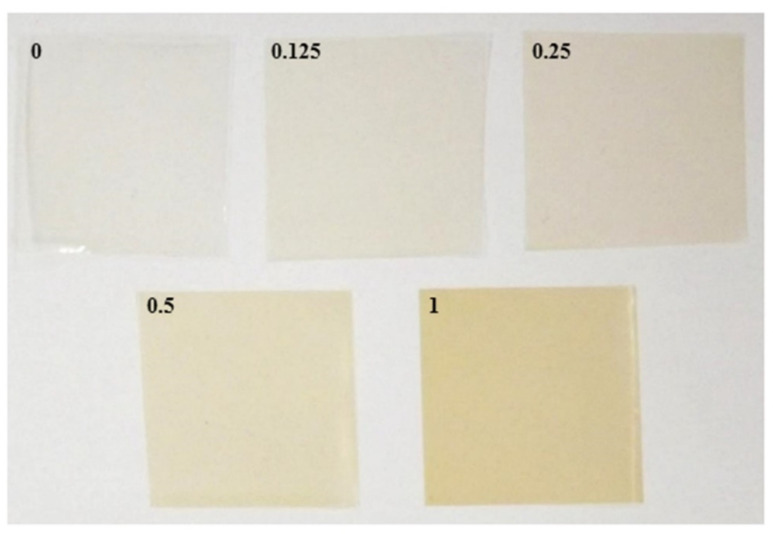
Film appearance of rice-starch-pectin films incorporated with green tea extract at different concentrations. The numbers designate concentrations of green tea extract (% *w/v*).

The appearance and color of rice-starch-pectin (RS-P) films at different concentrations of green tea extract (GTE) are shown in Figure 1 and Table 1, respectively. The control film (without GTE) was clear in appearance. As the GTE concentration increased, the films were darker, corresponding to the significant (*p* < 0.05) decrease in the L* value. The significant decrease in a* toward redness and increase in b* to yellowness were related to the reddish-yellow appearance of the developed GTE-enriched films. A similar report was observed in the GTE incorporated in chitosan film [33].

The light transmission of the RS-P-GTE film in the UV-Vis region (200–800 nm) is expressed in Table 2. The transmittance values of the developed RS-P-GTE film ranged from 0.10 to 89.33%. Incorporating GTE into the RS-P composite films reduced the light transmission through the film in the UV-Vis region. At higher GTE content, the reduction in transmittance was more pronounced. At 1.000% GTE, the RS-P-GTE composite film showed a low transmittance (0.01–25.14%) in the UV region (200–400 nm), indicating a strong UV light barrier. In addition, the addition of GTE decreased the light transmission in the visible region (400–800 nm) of the RS-P film. The RS-P control film (without GTE) exhibited the light transmittance in the visible region between 78.71 and 89.33%, while the RS-P-GTE composite film at 1.000% GTE displayed a reduction in the visible light transmittance in the range of 25.14–80.66%. The results suggested that adding GTE into the RS-P blend film absorbed the light in the range of the UV-Vis region. The natural pigments such as chlorophyll, lutein, and β-carotene [47], which are polyphenols in GTE that can scatter and refract the light on film surface [48], are possibly responsible for the decreasing transmittance in the RS-P-GTE composite film.

The transparency of the RS-P-GTE films, as shown in Table 2, is significantly (*p* < 0.05) decreased with the increase in GTE concentration, implying that the GTE incorporation led to the higher opacity of the resulting films. Therefore, the opaquer film could be beneficial as the packaging film for protecting the fatty foods where the light passage needs to be reduced.

### 3.2. Film Thickness and Mechanical Property

Table 3 illustrates the measured thickness and mechanical behavior of the developed film in terms of the tensile strength (TS) and elongation at break (EAB). Increasing the GTE content significantly (*p* < 0.05) increased the thickness of the RS-P-GTE composite film. A higher solid content may be responsible for the increase in film thickness. Furthermore, there is the possibility that the structures of the adjacent molecules interacted [49] through the hydrogen or noncovalent bonding between the reactive groups of polyphenols and polysaccharide chains of the rice-starch-pectin matrix [33,50,51] to form the inclusion complex during film preparation. Therefore, the association between the RS-P film matrix and the phenolic compounds through hydrogen bonds resulted in the complex inclusion of V-type amylose, a helix structure related to the inhibition of starch retrogradation [50] during the cooling process of the RS-P-GTE film preparation. In addition, the higher thickness of the RS-P-GTE composite films was found to be consistent with the reduced WVP of the developed RS-P-GTE blend film.

The tensile strength (TS) and the elongation at break (EAB) of the developed RS-P-GTE films are shown in Table 3. The results revealed that adding GTE decreased the TS and EAB of the RS-P-GTE composite film. This phenomenon can be explained by the large inclusion complex formed by H-bonding [52] of the RS-P matrix with the polyphenolic compounds, which resulted in a weaker film structure. Thus, these weak intermolecular bonds may contribute to the decreased flexibility [44,53] of the developed films.

### 3.3. Film Moisture Content and Film Solubility

The moisture content (MC) of the RS-P-GTE film is shown in Table 3. The control RS-P film (without GTE) possessed the highest MC (30.38 ± 0.36%). Moreover, the incorporation of GTE significantly (*p* < 0.05) reduced the moisture content of the RS-P-GTE composite films at increasing contents of GTE (28.58 ± 0.56% to 23.57 ± 2.77%). The decreased MC of the developed film with GTE could indicate the formation of the inclusion complex between the RS-P matrix and the tea polyphenolic compounds through hydrogen bonds [50,54,55]. These weak intermolecular interactions of the RS-P matrix and polyphenols were formed in competition with the water molecules, thus reducing the matrix’s availability to bind with water molecules [53], resulting in the reduction in moisture content of the developed film.

Film solubility (FS) in water denotes both film resistance and biodegradability of a film to water [56]. According to the results expressed in Table 3, the FS values of all developed films were not significantly altered. Therefore, the results suggested that the higher GTE concentration should be further investigated to determine the limit of GTE addition to maintaining the film stability as the low film solubility is ideal for food packaging applications.

### 3.4. Water Vapor Permeability

The water vapor permeability (WVP) of the RS-P-GTE composite film is illustrated in Table 3. The incorporation of GTE at below 1.000% *w/v* had not significantly altered the WVP of the developed film. However, at 1.000% *w/v* GTE addition, the WVP of the RS-P-GTE film started to significantly decrease (*p* < 0.05). At lower than 1.000% GTE, the formation of tea polyphenols and the RS-P matrix inclusion complex may not be profound. The hydrophobicity of the hollow complex was not intensified; thus, the passage of the water molecules can be carried out as usual. In contrast, as the GTE concentration reached 1.000% *w/v*, the resulting V-type inclusion complex of a single helix was strongly formed, which could possibly reduce the available hydrophilic exterior of the V-amylose helix [35,57]. In addition, the V-type amylose hydrophobic interior could also decrease the affinity to the water permeant, causing the tortuous path for water to travel through [58]. Thus, the resulting significant (*p* < 0.05) reduction in WVP at 1.000% *w/v* was observed. The result of no alteration in WVP was also observed in the GTE-chitosan composite film [33,50,51]. The potential of further reducing the WVP at higher GTE addition to the RS-P film brings about the possible application of the developed RS-P-GTE film as the food packaging where the moisture loss is concerned [59].

### 3.5. FTIR

**Figure 2:**
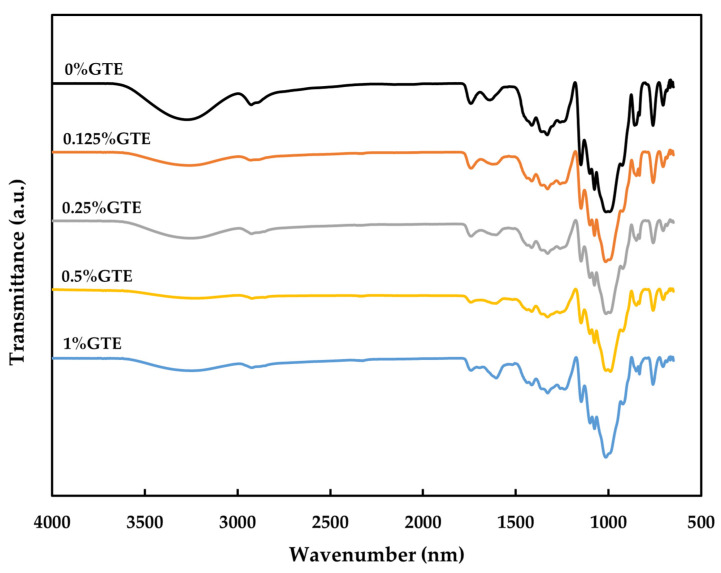
FT-IR spectra of RS-P composite films incorporated with green tea extract at different concentrations. Numbers denote the concentrations of GTE (%, *w*/*v*).

The FTIR spectra of various RS-P-GTE composite films are presented in Figure 2. FT-IR spectroscopy was used to reveal the interactions in the RS-P composite film when adding GTE. The spectra of RS-P-GTE composite films showed a similar pattern to the control RS-P blend film (without GTE), indicating no chemical reaction [60] in the functional groups of RS, P, and GTE. In addition, the RS-P blend and GTE may associate through the noncovalent interactions. All developed RS-P-GTE film samples displayed major bands at 3270 cm^–^^1^ (O-H stretching), 2926 cm^–^^1^ (C-H stretching), 1639 cm^–^^1^ (C=O stretching), and 1350 cm^–^^1^ (O-H bending, phenol). The RS-P-GTE composite films containing different GTE contents did not differ in the vibrational wavenumber for the major peaks. However, the -OH stretching peak of the RS-P-GTE films showed a slight shifting from 3270 cm^–^^1^ to around 3250 cm^–^^1^ after adding GTE with the decrease in sharpness and peak area, indicating that hydrogen bonds were formed between the RS-P matrix and the polyphenols [61]. The spectra shifts were associated with the inclusion formation of the RS-P matrix and the tea polyphenols, which affected the thickness, mechanical properties (i.e., TS and EAB), FS, and WVP of the developed films.

### 3.6. Determination of Total Phenolic Content

**Figure 3:**
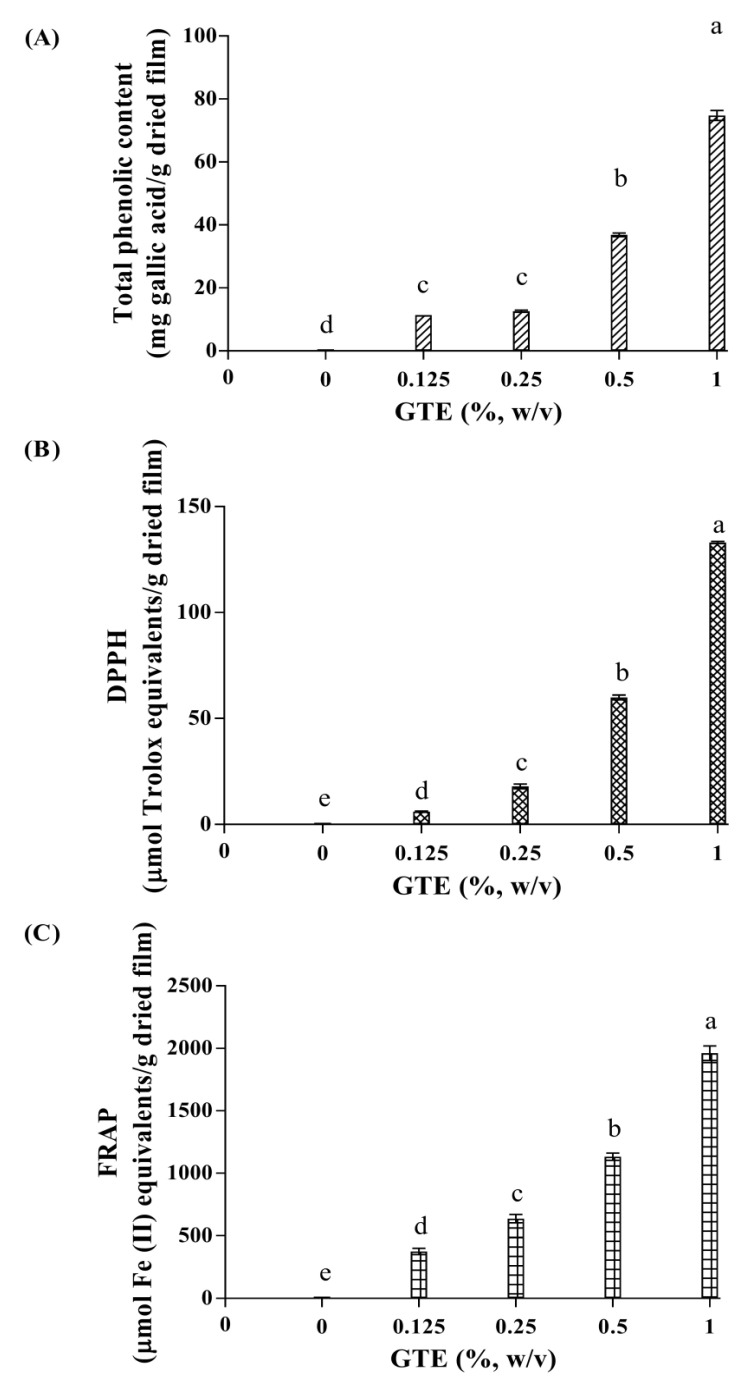
Total phenolic content (**A**), DPPH (**B**), and FRAP (**C**) of rice-starch-pectin films incorporated with green tea extract (GTE) at different concentrations. The numbers designate concentrations of green tea extract (%, *w*/*v*). Values (*n* = 3) are given as mean ± SD. Different letters indicate significantly different (*p* < 0.05).

The total phenolic content (TPC) of the RS-P-GTE composite film is shown in Figure 3A. The results revealed that the TPC values of all GTE-enriched films were significantly (*p* < 0.05) higher than that of the control RS-P film (without GTE). Green tea is a rich source of polyphenols, especially catechin, epicatechin, and epigallocatechin [23,42], which holds excellent antioxidant activity [43]. Thus, at 1.000% GTE content, the highest TPC was observed. A similar increasing TPC trend when increasing GTE incorporation was reported for GTE-enriched chitosan film [33].

### 3.7. Antioxidant Activities

The antioxidant activities (i.e., DPPH and FRAP assays) of all RS-P-GTE composite films were determined as illustrated in Figure 3B and Figure 3C, respectively. The control RS-P film (without GTE) had no DPPH radical scavenging activity and ferric reducing antioxidant power (FRAP). The results showed a significant (*p* < 0.05) increase in DPPH and FRAP antioxidant activities of the RS-P-GTE composite films as the GTE content increased, which corresponded to the increasing TPC in the RS-P-GTE films (Figure 3A). The TPC and other antioxidant agents [62] in GTE may be responsible for the antioxidant activities in the developed RS-P-GTE blend films. The increase in antioxidant activity in response to the increasing GTE was also reported by Siripatrawan and Harte [33]. The results suggested that the GTE could be used to tailor the antioxidant ability of the biocomposite active films for fatty food packaging applications [63].

### 3.8. Antimicrobial Activity

**Figure 4:**
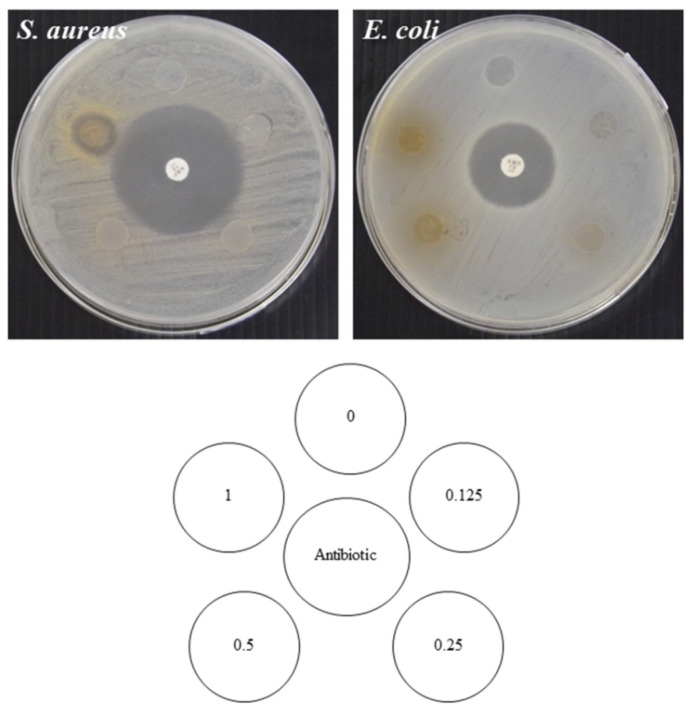
Antimicrobial properties of rice-starch-pectin films incorporated with green tea extract at different concentrations. The numbers designate concentrations of green tea extract, (%*w/v*).

The antimicrobial activities of the RS-P composite films enriched with different concentrations of GTE were tested against Gram-positive bacteria (*S*. *aureus*) and Gram-negative bacteria (*E*. *coli*), by the disc diffusion method in comparison with the control RS-P film (without GTE). As shown in Figure 4, the results revealed that all GTE-enriched composite films had no antimicrobial activity against *E*. *coli*. Furthermore, at lower than 1.000% GTE, the RS-P-GTE blend film had no antimicrobial activity against *S*. *aureus*. However, at 1.000% GTE, the inhibition zone against *S*. *aureus* was observed. In addition, an ability to inhibit Gram-positive bacteria was also reported in the GTE-nanofibrillated cellulose-starch composite film [64]. At lower than 1.000% GTE content, the RS-P matrix may interact with or hinder the migration or dissolution of the antimicrobial compounds present in the active food packaging [65], resulting in no inhibition zone observed against Gram-positive bacteria (*S*. *aureus*). In addition, the difference in cell wall structure [66] of these two bacterial strains also affected the antimicrobial activity of the tested film. According to Taylor et al. [67], the phenolic compound in GTE can inhibit the growth of both Gram-positive and Gram-negative bacteria. Thus, it is expected that the RS-P composite film with a GTE concentration higher than 1% (*w/v*) would show a stronger antimicrobial activity against Gram-positive bacteria and acquire some inhibition against Gram-negative bacteria.

## 4. Conclusions

The present study revealed that incorporating green tea extract (GTE) in the rice-starch-pectin (RS-P) composite film produced thicker, less transparent films in a reddish-yellow tone. In addition, the RS-P-GTE composite film solubility in water was stable. Additionally, a higher amount of GTE added to the RS-P film significantly (*p* < 0.05) decreased MC, TS, EAB, and WVP (at 1.000% GTE). The FT-IR spectra of the RS-P-GTE composite films indicated no significant changes in the functional groups in the RS-P composite film after GTE incorporation. However, there were intermolecular interactions between GTE and the RS-P film matrix. Furthermore, GTE significantly improved the antioxidant properties of the RS-P composite films. At the 1.000% GTE (*w/v*) addition, the RS-P composite film showed the inhibition against Gram-positive bacteria (*S*. *aureus* TISTR 764). The results suggested that the RS-P composite film with GTE incorporation exhibited the potential to be used as an active food packaging film. However, a study of the RS-P composite film with a higher range of GTE concentration enrichment along with an improvement in mechanical properties of the RS-P-GTE composite film may be further investigated to broaden its application in the food packaging industry.

## Figures and Tables

**Table 1:** The color values of rice-starch-pectin films incorporated with green tea extract at different concentrations.

GTE (% *w/v*)	L*	a*	b*	ΔE*
0.000	90.62 ± 0.84 ^a^	−1.13 ± 0.04 ^c^	4.39 ± 0.03 ^e^	5.63 ± 0.62 ^e^
0.125	88.80 ± 0.52 ^b^	−1.15 ± 0.02 ^c^	7.34 ± 0.31 ^d^	9.03 ± 0.37 ^d^
0.250	87.82 ± 0.60 ^c^	−1.13 ± 0.05 ^c^	10.44 ± 0.65 ^c^	12.11 ± 0.73 ^c^
0.500	86.91 ± 0.72 ^d^	−1.04 ± 0.04 ^b^	14.61 ± 0.69 ^b^	16.16 ± 0.83 ^b^
1.000	84.57 ± 0.57 ^e^	−0.52 ± 0.17 ^a^	24.48 ± 1.26 ^a^	26.11 ± 1.32 ^a^

Values are given as mean ± SD from *n* = 5. Different superscripts in each column are significantly different (*p* < 0.05). GTE: green tea extract (% *w/v*).

**Table 2:** Light transmission and transparency of rice-starch-pectin films incorporated with green tea extract at different concentrations.

GTE (% *w/v*)	Transmittance (%T) at Wavelength (nm)								Transparency *
	200	280	350	400	500	600	700	800	
0.000	0.10	42.50	71.66	78.71	84.72	87.28	88.60	89.33	3.29 ± 0.003 ^a^
0.125	0.04	1.06	37.48	64.37	75.55	79.34	81.22	82.56	3.21 ± 0.005 ^b^
0.250	0.04	0.03	19.32	56.08	74.07	78.95	81.12	82.49	3.17 ± 0.007 ^c^
0.500	0.03	0.01	8.71	46.26	71.47	77.50	79.82	81.23	3.15 ± 0.004 ^d^
1.000	0.02	0.01	0.79	25.14	65.71	75.63	78.52	80.66	3.09 ± 0.029 ^e^

* Values are mean ± SD (*n* = 3). Different superscripts in each column are significantly different (*p* < 0.05). GTE: green tea extract.

**Table 3:** Thickness, mechanical, physicochemical, and barrier properties of rice-starch-pectin films incorporated with green tea extract at different concentrations.

GTE (%, *w/v*)	Thickness (mm)	TS (MPa)	EAB (%)	MC (%)	FS (%)	WVP (×10^–11^ g m/m^2^ s Pa)
0.000	0.045 ± 0.001 ^d^	4.40 ± 0.12 ^a^	24.57 ± 1.57 ^a^	30.38 ± 0.36 ^a^	45.46 ± 4.15 ^a^	10.02 ± 0.41 ^a^
0.125	0.049 ± 0.001 ^c^	3.53 ± 0.26 ^b^	24.12 ± 1.89 ^a^	28.58 ± 0.56 ^ab^	46.43 ± 5.80 ^a^	9.57 ± 1.13 ^ab^
0.250	0.053 ± 0.001 ^b^	2.49 ± 0.26 ^c^	21.35 ± 3.07 ^b^	27.05 ± 0.69 ^bc^	47.13 ± 0.83 ^a^	9.09 ± 0.32 ^ab^
0.500	0.055 ± 0.001 ^b^	2.41 ± 0.09 ^c^	19.15 ± 1.52 ^bc^	25.58 ± 0.44 ^cd^	50.42 ± 6.30 ^a^	8.38 ± 1.59 ^ab^
1.000	0.061 ± 0.003 ^a^	2.12 ± 0.12 ^d^	18.53 ± 2.54 ^c^	23.57 ± 2.77 ^d^	52.52 ± 4.95 ^a^	7.94 ± 0.13 ^b^

Values are given as mean ± SD from *n* = 5 determination for thickness; *n* = 7 for determinations of TS and EAB; *n* = 3 for determinations of MC, FS, and WVP. Different superscripts in each column are significantly different (*p* < 0.05). GTE: green tea extract (%*w/v*), TS: tensile strength, EAB: elongation at break, MC: moisture content, FS: film solubility, WVP: water vapor permeability.

## Data Availability

The data presented in this study are available on request from the corresponding author.
